# Ultrasensitive and label-free biosensor for the detection of *Plasmodium falciparum* histidine-rich protein II in saliva

**DOI:** 10.1038/s41598-019-53852-5

**Published:** 2019-11-25

**Authors:** Gita V. Soraya, Chathurika D. Abeyrathne, Christelle Buffet, Duc H. Huynh, Shah Mukim Uddin, Jianxiong Chan, Efstratios Skafidas, Patrick Kwan, Stephen J. Rogerson

**Affiliations:** 1 Department of Medicine, The University of Melbourne, Royal Melbourne Hospital, Victoria, 3050 Australia; 2 Centre for Neural Engineering, The University of Melbourne, Carlton, VIC 3053 Australia; 3 Department of Electrical and Electronic Engineering, Melbourne School of Engineering, The University of Melbourne, Victoria, 3010 Australia; 4 The Peter Doherty Institute for Infection and Immunity, Victoria, 3000 Australia; 5 Department of Biochemistry, Faculty of Medicine, Hasanuddin University, Makassar, South Sulawesi 90245 Indonesia

**Keywords:** Medical research, Biomarkers

## Abstract

Malaria elimination is a global public health priority. To fulfil the demands of elimination diagnostics, we have developed an interdigitated electrode sensor platform targeting the *Plasmodium falciparum* Histidine Rich Protein 2 (*Pf*HRP2) protein in saliva samples. A protocol for frequency-specific *Pf*HRP2 detection in phosphate buffered saline was developed, yielding a sensitivity of 2.5 pg/mL based on change in impedance magnitude of the sensor. This protocol was adapted and optimized for use in saliva with a sensitivity of 25 pg/mL based on change in resistance. Further validation demonstrated detection in saliva spiked with *Pf*HRP2 from clinical isolates in 8 of 11 samples. With a turnaround time of ~2 hours, the label-free platform based on impedance sensors has the potential for miniaturization into a point-of-care diagnostic device for malaria elimination.

## Introduction

Malaria caused by intraerythrocytic *Plasmodium* parasites remains a significant public health threat. *P. falciparum* is responsible for most severe malaria illness and almost all deaths^1,2^ which occur mainly in young children in the World Health Organization’s African Region^3^.

In recent years the burden of malaria has decreased, through improvements in treatment and prevention. From 2010 to 2015, global malaria incidence and numbers of deaths decreased by 21% and 29% respectively^3^. With the reduction of transmission rates and the malaria burden, elimination agendas have been pushed with the aim to end local transmission of the disease in at least 35 countries by the year 2030^4^.

Clinical malaria diagnosis relies on light microscopy (LM) for visual confirmation of parasites or rapid diagnostic tests (RDTs) to detect parasite antigens using lateral-flow technology^5^. A common RDT target is the *P. falciparum* histidine-rich protein II (*Pf*HRP2) antigen, a multiplet protein^6,7^ produced exclusively in the *P. falciparum* parasite cytoplasm and exported to the parasitized erythrocyte membrane^8^. The *Pf*HRP2 protein is readily detectable in whole blood, serum, plasma, urine^9^ and saliva^10,11^ samples of infected patients.

For malaria elimination, current diagnostics need to be adapted to detect increasing numbers of asymptomatic parasite carriers, with the essential and desirable target sensitivity in the context of population screening at 20 parasites/µL blood and ≤5 parasites/µL blood respectively^12^. Current RDTs however have a relatively low sensitivity. In a study assessing the relationship between antigen concentration and parasite density, a minimum of 4 ng/mL of *Pf*HRP2 was required to obtain 95% positive results in a panel of malaria-infected blood samples with a parasite density of 200 parasites/µL^13^. A more recent study^14^ assessing the current best-in-class *Pf*HRP2 RDTs according to the WHO-Foundation for Innovative New Diagnostics panel^15^ found an analytical sensitivity of 0.8 ng/mL. It can be inferred from these studies that to achieve the target sensitivity for elimination, *Pf*HRP2 diagnostic tests need to be 1–2 logs lower than achievable by current RDTs.

While *Pf*HRP2 ELISAs^16–18^, PCR^19,20^ and loop-mediated isothermal amplification (LAMP)^21^ assays have shown superior sensitivity to RDTs for detection of low-density infections, the platforms are slow and technically complex assays typically performed by highly-skilled technicians in centralized laboratories. Implementation of these methods is therefore impractical in the low-resource settings of many countries striving for malaria elimination.

Additionally, detection of *Pf*HRP2 in saliva is gaining substantial interest due to ease of collection, lower biohazard risk, and higher likelihood of testing compliance particularly in communities where blood collection poses cultural objections^22,23^. Although salivary *Pf*HRP2 has been evaluated for the detection of low to high-density *P. falciparum* parasitemias^10^ no RDTs have been developed for this purpose, due to the lack of sensitivity. An in-house ELISA assay developed for detection of salivary *Pf*HRP2 had a sensitivity of 0.17 pg/mL^7^, but the laboratory-based nature of the platform hinders effective field implementation.

Impedimetric biosensors are promising options to help close current diagnostic gaps, due to their high sensitivity, low cost, and amenability to miniaturization. Table 1 summarises previous reports on the development of ultrasensitive *Pf*HRP2 biosensors. The biosensors were used in direct or sandwich immunoassay formats to target *Pf*HRP2 protein in various sample matrices. To achieve high-level sensitivity, most of these sensors required additional labeling and signal amplification, which in the long run may incur additional costs during miniaturization.

**Table 1:** Comparison between previously developed *Pf*HRP2 immunosensors.

*Pf*HRP2 Immunosensors					
Assay Format	Assay Time	Labelling or Signal Amplification	Immunosensor Type	Lowest concentration of *Pf*HRP2 detected	Reference
Sandwich immunoassay	~ 30 minutes	ALP	SPEs with MWCNT and AuNPs characterized with cyclic voltammetry	8 ng/mL in DEA buffer	^43^
Direct immunoassay	~ 1 hour	Label-free	Piezoelectric sensor characterized with cyclic voltammetry and EIS	12 ng/mL in Tris buffer	^37^
Direct immunoassay	NA	Redox couple	Faradaic EIS using Cu-doped ZnO electrospun nanofibers	6 ag/ml in PBS buffer	^44^
Sandwich immunoassay	1 minute	CNF grown on NMBs	Immunochromatography + resistivity measurements	0.01 ng/mL in PBS buffer	^45^
Sandwich immunoassay	>2 hours	HRP	Amperometric; Electrochemical magneto immunosensor coupled with magnetic nanoparticles	0.36 ng/mL in spiked serum	^46^
Sandwich immunoassay	>2 hours	HRP and AuNP	Cyclic voltammetry on SPEs	36 pg/mL in milk PBS buffer; 40 pg/mL in spiked Serum	^38^
Sandwich immunoassay	~1 hour	MB, Ru(NH_3_)_6_^3+^ and TCEP	ECC redox cycling scheme for signal amplification on 3-electrode sensor	10 fg/mL in spiked plasma; 18 fg/mL in spiked whole blood	^39^
**Direct Immunoassay**	**1–2 hours**	**Label-free**	**Non-faradaic IDE Sensors**	**2.5 pg/mL in PBS buffer; 25 pg/mL in spiked saliva**	**This work**

ALP = alkaline phosphatase, SPE = screen-printed electrodes, MWCNT = multiwall carbon nanotube, DEA = diethanolamine buffer, EIS = electrochemical impedance spectroscopy, Cu = copper, ZnO = zinc oxide, PBS = phosphate buffered saline, CNF = carbon nanofiber, NMBs = glass microballoons, HRP = horseradish peroxidase, AuNP = gold nanoparticles, MB = methylene blue, Ru(NH_3_)_6_^3+^ = hexaamineruthenium(III) chloride, TCEP = tris(2-carboxyethyl)phosphine hydrochloride, ECC = electrochemical-Chemical, IDE = interdigitated electrodes.

In this study, we aimed to develop an interdigitated electrode (IDE) sensor for impedimetric detection of *Pf*HRP2 at low concentrations suitable for malaria elimination, with a focus on the utilization of saliva as sample medium. Compared with other technologies, the IDE sensor geometry has demonstrated high level of sensitivity and specificity for label-free detection of various targets including nucleic acids^24–27^, cells^28–30^, and proteins^31–33^. Here, the detection platform utilized anti-*Pf*HRP2 monoclonal antibodies (MAbs) immobilized on sensor surface as capture probes towards circulating *Pf*HRP2 protein. The application of periodic small AC voltage allowed measurement of the sensor impedance, defined as a complex of the circuitry’s resistance and capacitance. Specific detection of surface-bound *Pf*HRP2 on the sensor was achievable through frequency-dependent characterization of the impedimetric changes. The platform demonstrates promising ultrasensitive detection of *Pf*HRP2 protein in saliva. Fabricated using low-cost techniques, the platform is amenable to future automatization and miniaturization for point-of-care applications.

## Results

### Sensor preparation and platform development

**Figure 1:**
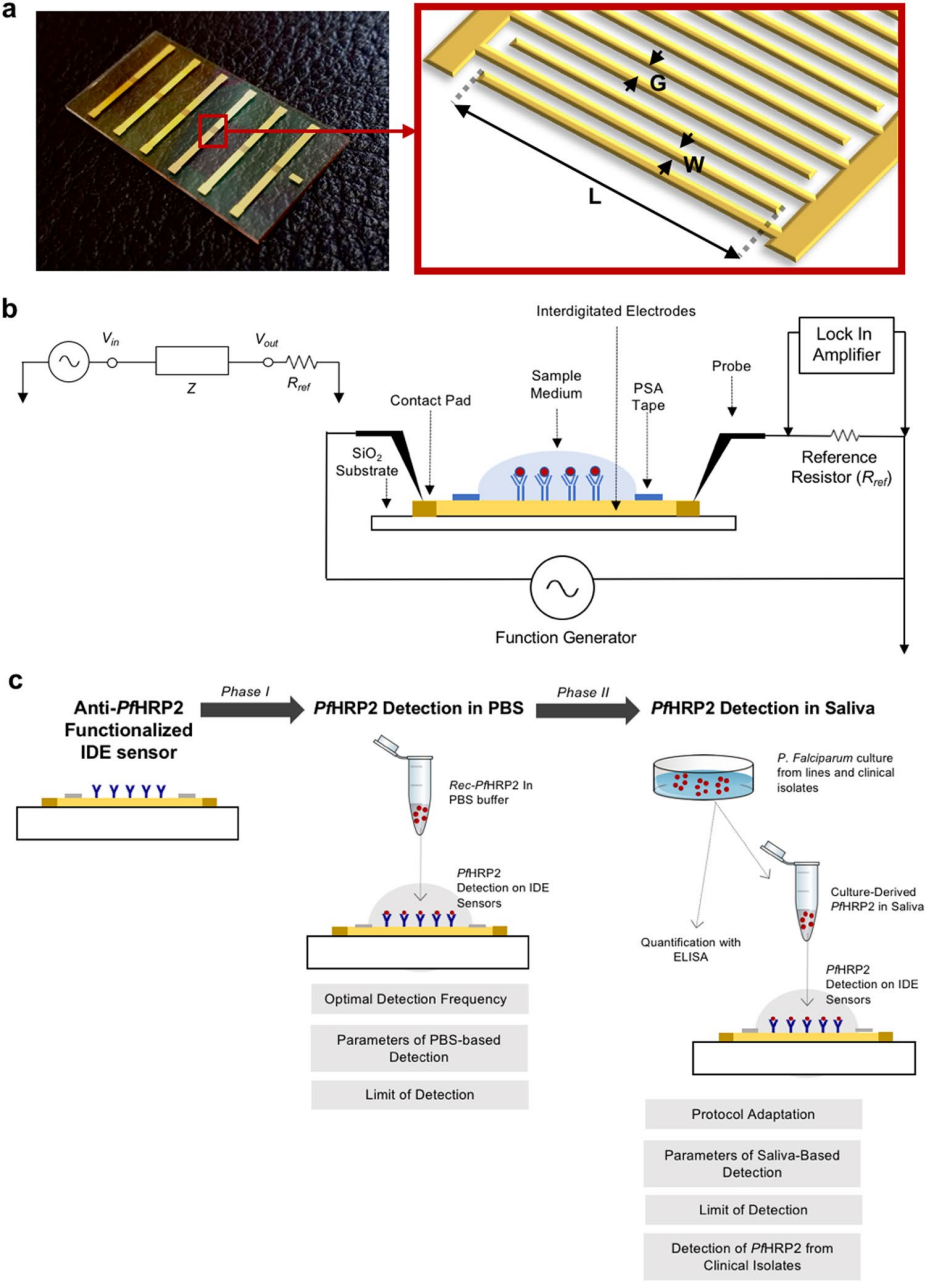
Sensor preparation and platform development. (**a**) Fabricated IDE sensor array and schematic of the IDE sensing area geometry. L = length, W = width, and G = gap of the working electrode. (**b**) Circuit model and measurement setup used for characterization of *Pf*HRP2 capture. The sensor was set up as a resistor (R) in series with a capacitor (C), with the associated input voltage (*V*_*in*_), output voltage (*V*_*out*_) and reference resistor (*R*_*ref*_) labeled accordingly. The cross-sectional view of the measurement setup depicts detection of *Pf*HRP2 protein in a sample medium with the sensing area designated by pressure-sensitive adhesive (PSA) tape. The IDE sensor is composed of interdigitated electrodes with adjoining electrode contact pads, and borosilicate glass substrate. Probe electrodes were placed on the contact pads to deliver excitation current to, and to measure electrical signals from, the sensors. (**c**) The two phases of platform development.

IDE sensors were fabricated using UV-lithography on borosilicate glass wafers as previously described^27^, with modification. An additional SiO_2_ layer was deposited at a thickness of 25 nm. Each sensor consisted of paired electrode arrays with finger length (L) of 980 µm, finger width (W) of 8 µm and gap width (G) of 8 µm (Fig. 1a). The sensors were functionalized according to our established protocol^30,34^ by covalent immobilization of anti-*Pf*HRP2 antibodies on the surface, followed by blocking to reduce non-specific binding to the antibodies. Optimization results of capture antibody concentration are provided in Supplementary Figure 1.

Impedance of the sensors was measured using our previously established circuit setup^30,34^ shown in Fig. 1b, in which the sensor is represented as a resistor (R) in series with a capacitor (C) connected in series with a 1 kΩ reference resistor (*R*_*ref*_). A function generator provided the input sinusoidal AC excitatory signal (*V*_*in*_) at 20 mV peak-to-peak voltage (*V*_*pp*_) at multiple frequencies (1,10, 20, and 40 kHz). Sensors were measured in wet-state (in PBS 1×) at time points T1 (baseline measurement obtained before sample incubation) and T2 (after sample incubation and washing) to detect binding associated change occurring between the two points. A lock-in amplifier recorded the amplitude of the output voltage (*V*_*out*_) and phase across the *R*_*ref*_, which was used for the acquisition of frequency (*ω*) dependent impedimetric parameters including impedance magnitude (*Z*), capacitance (*C*), and resistance (*R*) using the following equations^34^

1 $${V}_{out}/{V}_{in}={R}_{ref}/(Z+{R}_{ref})$$

2 $$Z=R-{\rm{j}}/(wC)$$

Development of the sensor platform was divided into two phases (Fig. 1c). In Phase I, phosphate buffered saline (PBS) samples spiked with *Pf*HRP2 were used. The optimal detection frequency was determined, and parameters for specific *Pf*HRP2 detection were established and used to obtain the analytical sensitivity. The protocol was then applied in Phase II in which saliva samples spiked with *Pf*HRP2 derived from cultured cell lines were used. Optimal parameters for saliva-based detection were determined to obtain the sensitivity. Specificity was then assessed using a panel of saliva samples spiked with isolate-derived *Pf*HRP2.

### Phase I - Detection of PfHRP2 protein in PBS buffer

**Figure 2:**
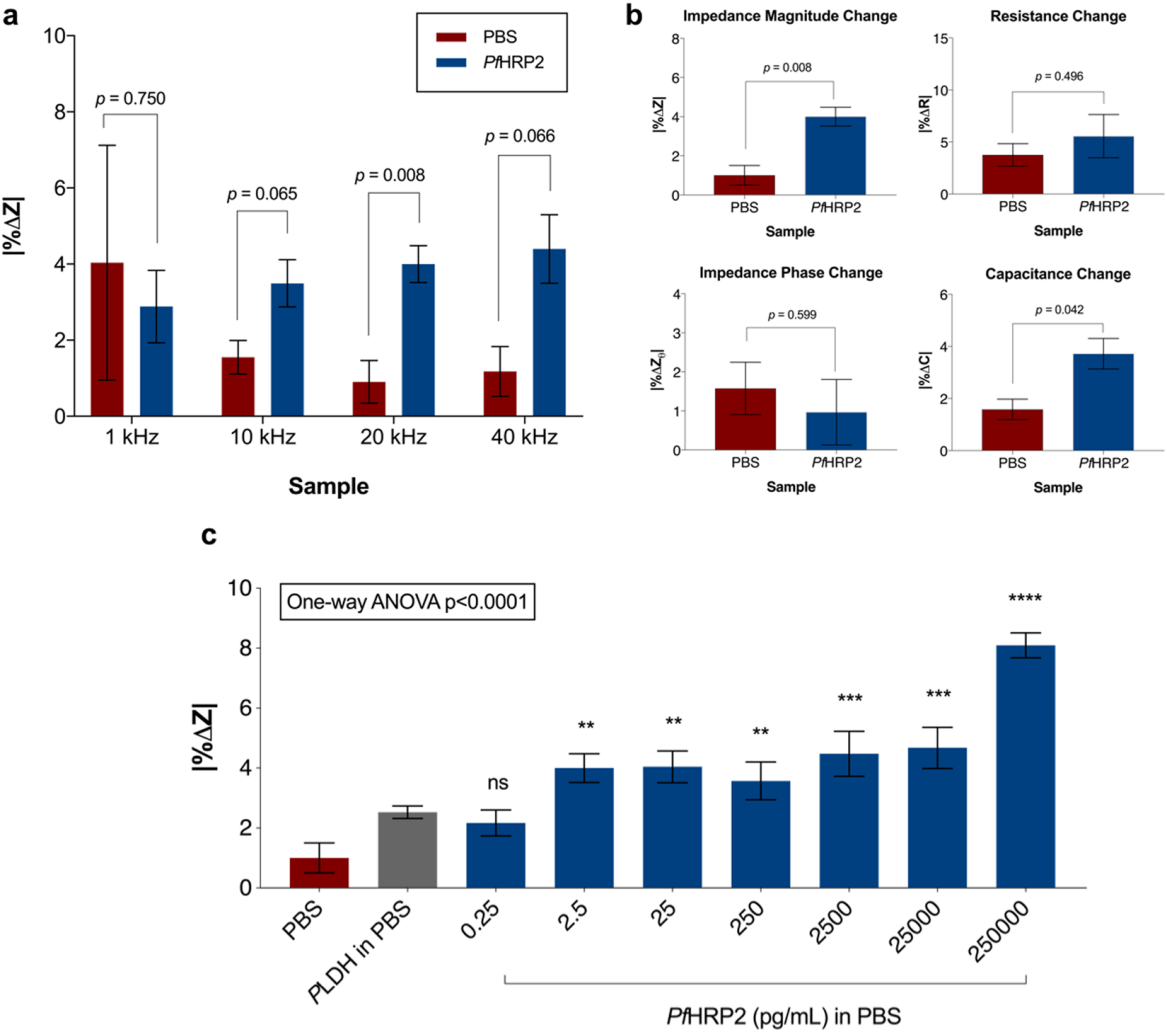
Detection of *Pf*HRP2 in PBS buffer using the sensors. (**a**) Optimal frequency for the detection of *Pf*HRP2 (2.5 pg/mL) in circuit setup. (**b**) Parameters for *Pf*HRP2 (2.5 pg/mL) detection in PBS buffer at 20 kHz frequency. (**c**) Sensor response towards various concentrations of *Pf*HRP2 protein in PBS buffer. Change in impedance associated with serial dilution of *Pf*HRP2 from 0.25 pg/mL to 250 ng/mL was measured at the 20 kHz optimal frequency. Figures show mean ± standard error of the mean (SEM), (**a,b**) *n* = 3 sensors per group, (**c**) *n* = 4–5 sensors per concentration, *p*-values calculated with Welch’s two-tailed t-test (**a,b**) or by one-way ANOVA followed by Dunnett’s multiple comparisons test with blank PBS used as the comparator (**c**). *p ≤ 0.05, **p ≤ 0.01, ***p ≤ 0.001, ****p ≤ 0.0001, *ns* = not significant. Red bars = sensors incubated in PBS buffer, blue bar = sensors incubated in *Pf*HRP2. All incubations were performed for 1 hour within a parafilm-wrapped petri dish to avoid sample evaporation.

Among the range of excitation frequencies applied (1 kHz to 40 kHz), 20 kHz was found to elicit the most significant difference in the change in impedance magnitude between PBS samples with and without spiked *Pf*HRP2 (Fig. 2a). This frequency was used for all subsequent experiments. The impedimetric parameters (impedance magnitude, impedance phase, resistance and capacitance) were also compared in their ability to differentiate PBS samples with and without spiked *Pf*HRP2, and the change in impedance magnitude was found to be the optimal parameter for this purpose (Fig. 2b).

The finding that frequency-dependent characterization of *Pf*HRP2 binding is optimal within the frequency range of 10–100 kHz is consistent with similar previous studies^30,35^, and supports the notion that the signal is primarily due to the changes in bulk medium conductivity as a result of dipole accumulation^36^ from the surface-bound *Pf*HRP2.

The optimal excitation frequency and impedimetric parameter were then used to determine the limit of *Pf*HRP2 detection in PBS medium. The limit of detection, defined as the concentration at which the test sample exhibited a signal change significantly distinct from the blank, was found to be 2.5 pg/mL (Fig. 2c). Starting at that *Pf*HRP2 concentration, a significant increase in the impedance signal was observed, indicating the sensor response towards the solid-state capture. No signs of saturation or prozone effect were observed at the higher end of the tested concentrations. This detection limit was concordant with our previous finding that the IDE sensors were able to detect another protein at the minimum concentration of 2.9 pg/mL^33^.

The Phase I results provided proof-of-concept data for ultrasensitive *Pf*HRP2 protein detection using the label-free electrical biosensor platform. The detection limit was 3 logs lower than that of the average RDTs (~ 4 ng/mL)^13^, and is also lower than previously developed label-free electrical biosensor for *Pf*HRP2, which reported a sensitivity of 12 ng/mL^37^.

### Phase II - Detection of PfHRP2 protein in saliva

Next, the ability of the platform to detect culture-derived *Pf*HRP2 in human saliva samples was evaluated to support future real-world applications. Saliva can be collected non-invasively, making it an ideal biospecimen for population screening. The protocol in Phase I was modified for use in saliva samples owing to differences in the ionic composition and protein content between PBS and saliva, and the need to reduce degradation of *Pf*HRP2 by protease enzymes present in saliva^7^. The modification included more stringent blocking protocol (Supplementary Figure 2), longer incubation time of 2 hours (Supplementary Figure 3**)**, sample pretreatment with protease inhibitor and re-characterization of the impedimetric detection parameters. The optimized parameters were then used to identify the limit of detecting *Pf*HRP2 in saliva and to validate the protocol using saliva samples spiked with *Pf*HRP2 expressed by different *P. falciparum* isolates.

**Figure 3:**
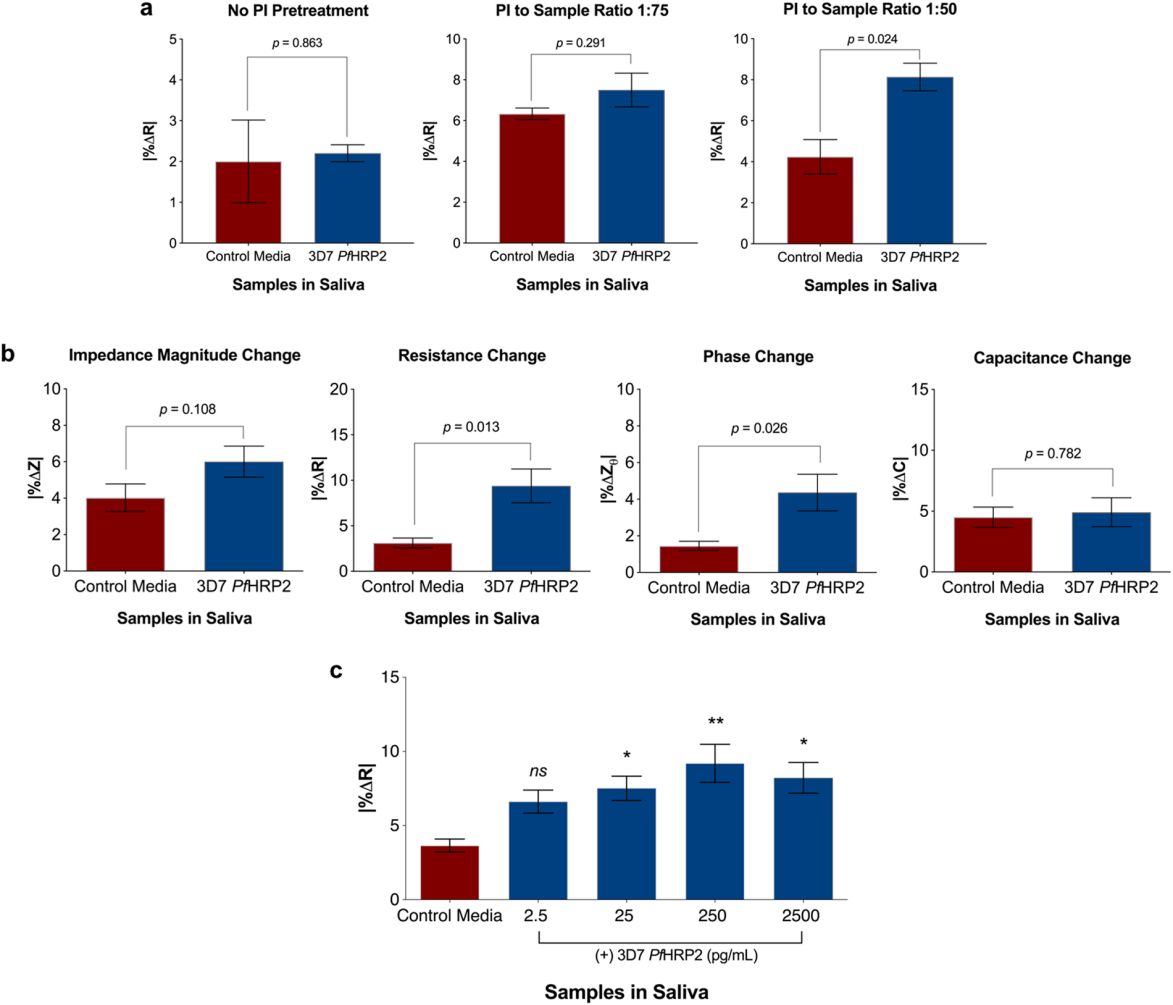
Detection of *Pf*HRP2 protein in saliva. (**a**) Optimization of protease inhibitor (PI) pretreatment for saliva samples. (**b**) Change in sensor resistance is the optimal impedimetric parameter for detection of *Pf*HRP2 (25 pg/mL) protein in saliva. Red bar = sensors incubated in unspiked saliva, blue bar = sensors incubated in 2.5 pg *Pf*HRP2 per mL of saliva. Bars represent mean ± SEM, *n = *7 sensors per group, *p*-values calculated with Welch’s two-tailed t-test. (**c**) Sensor response towards various concentrations of *Pf*HRP2 protein in saliva. A serial dilution of 2.5 pg/mL to 2.5 ng/mL *Pf*HRP2 protein in saliva was measured. Bars represent mean ± SEM, *n* = 7–8 sensors per concentration. Statistical analysis by one-way ANOVA followed by Dunnett’s multiple comparisons test with blank saliva used as the comparator. *p ≤ 0.05, **p ≤ 0.01, *ns* = not significant. All incubations were performed for 2 hours within a parafilm-wrapped petri dish to avoid sample evaporation, and all measurements were performed at 20 kHz frequency.

Unlike findings in Phase I that used PBS, change in sensor resistance was found to be a more specific parameter in differentiating saliva samples with and without spiked *Pf*HRP2 (Fig. 3b). The finding that optimal differentiation is parameter-specific is one of the advantages of impedance-based measurements, as it permits optimal detection despite differences in ionic composition across sample types.

The adapted parameters were used to determine the lowest limit of *Pf*HRP2 detection, which was found to be 25 pg/mL (Fig. 3c). Starting at this concentration of *Pf*HRP2, the change in sensor resistance was significantly different between the positive sample and the negative (unspiked) sample. In contrast to the PBS-based dynamic range assessment, signal saturation was observed earlier in the saliva concentration curve, at 2.5 ng/mL *Pf*HRP2. This saturation, which resulted in a less optimal concentration-dependent response, is most likely due to the presence of interfering proteins in the saliva matrix compared to the PBS, which may have been the cause of reduction in binding magnitude. The presence of multitude of substances in saliva can result in failure of antibody and ligand complex formation on the sensor surface, which may contribute to the reduced dose-dependent effect.

**Figure 4:**
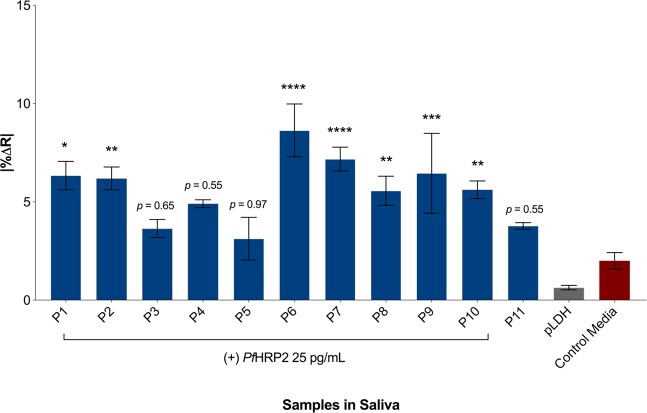
IDE sensor performance for *Pf*HRP2 detection in spiked-saliva samples. Saliva samples were spiked with culture media and *Pf*HRP2 (blue bars, *n* = 3–4 sensors per group) or *P*LDH (grey bar, n = 3 sensors) at a uniform concentration of 25 pg/mL, and blank (un-spiked) (red bar, *n* = 11 sensors). P_1–9_ were saliva samples spiked with *Pf*HRP2 in culture supernatants of clinical malaria isolates, P_10–11_ were spiked with *Pf*HRP2 isolated from supernatants of laboratory lines 3D7 and CS2. Applied frequency 20 kHz, 2-hour incubation within a parafilm-wrapped petri dish to avoid sample evaporation.. Bars represent mean ± SEM. Statistical analysis by one-way ANOVA followed by Dunnett’s multiple comparisons test with blank saliva used as the comparator. *p ≤ 0.05, **p ≤ 0.01, ***p ≤ 0.001, ****p ≤ 0.0001.

To further validate the protocol for real-world implementation, 11 saliva samples spiked with *Pf*HRP2 (samples P_1–9_ are *Pf*HRP2 present in supernatants of clinical parasite isolates, P_10–11_ are *Pf*HRP2 isolated from culture supernatants of 3D7 and CS2 laboratory adapted parasites lines) at a concentration of 25 pg/mL were incubated with the sensor platform. Among the 11 samples tested, 8 resulted in significantly higher change in resistance (%Δ*R*) compared to the un-spiked saliva sample and the sample spiked with *Plasmodium* lactate dehydrogenase (*P*LDH) (Fig. 4). The variations among the sensor signals from the different *Pf*HRP2 samples might be attributed to the differences in *Pf*HRP2 protein size since previous characterizations have shown the protein to be of multiple bands ranging from 50–80 kDa^7^, although this assumption will need to be assessed further.

## Discussion

The sensor platform developed in this study represents a highly sensitive impedimetric sensor capable of label-free detection of surface-bound *Pf*HRP2. Promising sensitivity was demonstrated with a *Pf*HRP2 detection limit of 2.5 pg/mL in PBS, an order of 3 logs lower than current *Pf*HRP2 RDTs. Furthermore, the study also demonstrated the feasibility of *Pf*HRP2 detection in saliva with a detection limit of 25 pg/mL, in 8 out of 11 tested samples. Specific detection was achieved using an optimized, constant excitation frequency of 20 kHz across both PBS and saliva samples.

Differences in assay sensitivity are commonly observed in the development of *Pf*HRP2 immunosensors as previous studies have also reported the variations in assay performance among various matrices^38,39^, with few studies examining saliva samples. Adaptation of the platform for other types of biospecimens, such as whole blood or plasma, would require re-optimization of impedimetric parameters, incubation time, and blocking protocol due to the differences in the ionic properties and protein content of different specimen types.

Saliva-based *Pf*HRP2 detection is an attractive and potentially cost-effective testing approach. To date, quantitative data on salivary *Pf*HRP2 in cases of low-density parasitemia is lacking, as previous salivary *Pf*HRP2 quantification using commercial^10^ or in-house developed^7^ ELISAs has been limited to testing of symptomatic individuals. Although there is currently no consensus regarding the correlation between saliva *Pf*HRP2 and parasitemia, the sensitivity shown by the platform is adaptable towards the range of salivary *Pf*HRP2 detected using the laboratory-based ELISA (17–1167 pg/mL)^7^. To futher guide the development of salivary *Pf*HRP2 diagnostics for elimination and screening purposes, more research is required to study the concentration of salivary *Pf*HRP2 protein in asymptomatic versus symptomatic individuals, and in low versus high density parasitemias. Additionally, more work is required on assessing the effect of patient conditions such as dehydration or hormonal fluctuations on the sensor readings, as minor physiological fluctuations can affect the saliva sample matrix.

Future work on the platform will focus on the miniaturization of sensors and integration of the platform with microfluidics. The lock-in amplifier readout technique has good miniaturization potential, with setup possible within small-scale portable devices^40,41^. Miniaturization and integration of the sensor platform has the potential to (1) reduce variation due to automatization of the electrical reading and washing steps, (2) reduce the required incubation time by avoiding the diffusion limited process of static incubation, and (3) improve efficiency due to reduction in the reagent and sample volume. Additionally, future work can also be geared towards adaptation of the IDE sensor platform to detect other proteins relevant for malaria diagnostics, including *P*LDH, and aldolase.

Ultrasensitive diagnostics are considered to play an important role in the global effort towards malaria eradication. Efforts continue to strive for a balance between assay performance, cost-effectiveness, and practicality. We have developed the *Pf*HRP2 IDE sensor platform with these factors in mind. Our study has provided proof of concept that the platform may be a potential technology to help achieve this goal.

## Methods

### Sample preparation

In Phase I, recombinant *Pf*HRP2 protein (CTK Biotech, California, USA) was suspended in PBS 1× buffer. Antigen quantification was performed using commercial *Pf*HRP2 ELISA (Cellabs Pty. Ltd., Brookvale, New South Wales, Australia). An additional negative control was prepared by suspending 2.5 pg of *P*LDH protein (CTK Biotech, California, USA) in 1× buffer.

In Phase II, detection was performed in saliva samples spiked with *Pf*HRP2 antigen harvested from *in vitro* culture supernatants. Culture specimens used included the *P. falciparum* laboratory lines 3D7 and CS2, in addition to 9 clinical isolates from Papua New Guinean (PNG) and Malawian children with malaria. The clinical isolates were collected as part of projects approved by the PNG Institute of Medical Research Institutional Review Board (IRB Number 136 1103) and the Medical Research Advisory Committee of the PNG Health Department (MRAC 137 Number 11.12) or by the College of Medicine Research Ethics Committee in Malawi (11/14.1566). Parents or guardians of infected children gave informed consent before venous blood was collected. The studies complied with the ethical standards of the Helsinki Declaration.

All specimens were cultured for 36 hours, to obtain samples at 6% parasitemia at mature trophozoite stage. Spent culture medium supernatants were collected. Control medium was prepared similarly by incubating medium with uninfected erythrocytes. Supernatants were stored at −80 °C and used to quantify *Pf*HRP2 by ELISA, and to detect HRP2 using the sensors. Control medium was used to spike negative saliva controls.

To prepare spiked saliva samples, unstimulated fresh saliva was collected and subjected to mechanical filtration to remove residues and mucus (Corning^®^ 0.2 µm filter). Protease inhibitor 100× (P8340 Protease Inhibitor Cocktail, Sigma Aldrich, Missouri, USA) was added immediately followed by the culture-harvested *Pf*HRP2 antigens. The *Pf*HRP2 samples (*n* = 11) were diluted using saliva to the final concentration of 25 pg/mL. Negative control blank samples (*n* = 11) were prepared by diluting the control medium in saliva to match the dilution of each *Pf*HRP2 sample. An additional negative sample was prepared using *P*LDH protein spiked saliva at 25 pg/mL. Spiked samples were immediately added to sensor after baseline electrical readings.

### Sensor fabrication

Fabrication was performed at the Melbourne Centre for Nanofabrication (MCN). Wafers of BOROFLOAT^®^ borosilicate glass (UniversityWafer, Massachusetts, USA) were cleaned with isopropyl alcohol, dried, and then coated with hexamethyldisilazane (MicroChemicals GmbH, Ulm, Germany) and AZ1512HS (MicroChemicals GmbH, Ulm, Germany) photoresist. A chrome mask of the sensor design was applied on the substrate followed by UV exposure (75 mJ/cm^2^). After development, a thin film of chrome (5 nm), gold (100 nm), and titanium (5 nm) was deposited on the substrate, followed by a lift-off process to reveal the IDE pattern. This was followed by an addition of a thin layer (25 nm) of SiO_2_ using e-beam evaporation (Intlvac Nanochrome^TM^ II, Colorado, USA).

### Sensor functionalization and antibody immobilization

The sensors were cleaned using a wash of acetone, isopropyl alcohol, and H_2_O, and then dried under N_2_ gas. Organic contaminants were eliminated using plasma treatment (PE-25 Plasma Etch, Nevada, USA) with argon (75%) and oxygen (25%) for 5 min at 50 W power and 30 cc/min flow rate. Sensors were silanized in 2% APTES (Sigma Aldrich, Missouri, USA) solution for 1 hour, followed by 3× 5-min washes in 100% ethanol with gentle shaking. Sensors were then immersed in 2.5% glutaraldehyde solution (Sigma Aldrich, Missouri, USA) for 2 hours to allow development of the bifunctional cross linker. After washing for 3× 5-min in PBS 1× with gentle shaking, sensors were dried under N_2_ gas. Both APTES and glutaraldehyde solutions were filtered prior to use with Corning^®^ 0.2 μm pore size filters to remove impurity. Following functionalization, a 2 mm diameter incubation area was established using medical grade pressure sensitive adhesive tape (Adhesive Research, Pennsylvania, USA).

Anti-*Pf*HRP2 IgG MAb receptor (AB-0445, Vista Diagnostics International, Washington, USA) at a volume of 15 µl (50 µg/mL) was applied on the modified sensing area and incubated in a humid chamber at 4 °C overnight. Sensors were then washed with PBS 1× for 5 min and blocked for 30 min using either 5% ethanolamine (Sigma Aldrich, Missouri, USA) for Phase I or 5% ethanolamine and 2.5% normal goat serum mix (Sigma Aldrich, Missouri USA) for Phase II.

### Data processing and statistics for PfHRP2 detection

Acquisition of impedance properties from the lock-in amplifier *V*_*out*_ and phase output was calculated based on Eqs. 1 and 2 using MATLAB. The baseline measurement obtained before sample incubation (T1) were first evaluated for assessment of sensor quality, then T1 and T2 (after sample incubation and washing) values for each parameter were processed in Microsoft Excel to obtain the percentage changes in impedance magnitude (%*∆Z* = ABS Z_T2_/Z_T1_), impedance phase (%*∆Z*_*θ*_), resistance (%*∆R*), and capacitance (%*∆C*). The absolute percentage change in impedance (|%*∆Z*|), resistance (*|*%*∆R*|), and capacitance (|%*∆C*|) were then used to assess the sensor performance statistically.

GraphPad PRISM 7 was used to generate all plots and perform statistical analysis, and *p*-values ≤ 0.05 were considered statistically significant. To implement quality control on the sensors, a baseline outlier test was performed using the regression and outlier test (ROUT)^42^ on all derived T1 values, with the maximum false discovery rate (Q) set to 1%. An example of the PRISM ROUT test performance can be seen in Supplementary Table 1 and Supplementary Figure 4. Sensors with baselines excluded using the ROUT test were not included in the rest of the analysis.

In Phase I, Welch’s two-tailed t-test was used to compare the percentage change in impedance magnitude (%∆*Z*) between blank and test sensors. A serial dilution of *Pf*HRP2 in PBS 1× buffer was incubated on the sensors, and the %∆*Z* obtained was assessed using one-way ANOVA with Dunnett’s multiple comparisons test. Detection limit is defined as the lowest tested concentration showing statistically significant difference from blank sensor reading.

In Phase II, Welch’s two tailed t-test was used to determine the optimal sample pretreatment and detection parameter in saliva. The optimized parameters were used to determine the detection limit in the same manner as in Phase I, using the optimized parameter for saliva (%*∆R*). Platform performance was then assessed in a panel of *Pf*HRP2-spiked saliva using the Dunnett’s multiple comparisons test to determine the degree of differentiation against the un-spiked saliva control.

## Supplementary information

Supplementary Information on the Development of an ultrasensitive and label-free biosensor for the detection of Plasmodium falciparum histidine-rich protein II in saliva

## Data Availability

Relevant data are available from the authors on request.

## Code availability

The codes used for impedimetric calculations are available on request from the authors.
