# Supplementary material for: Ultrasensitive and label-free biosensor for the detection of Plasmodium falciparum histidine-rich protein II in saliva
Source: Sci Rep. 2019 Nov 25;9:17495. doi: 10.1038/s41598-019-53852-5 (PMC6877566; doi:10.1038/s41598-019-53852-5)
Supplement: Supplementary file 1 — Supplementary Information on the Development of an ultrasensitive and label-free biosensor for the detection of Plasmodium falciparum histidine-rich protein II in saliva [file 41598_2019_53852_MOESM1_ESM.docx]

**SUPPLEMENTARY INFORMATION**

**TITLE**

Ultrasensitive and label-free biosensor for the detection of *Plasmodium falciparum* histidine-rich protein II in saliva.

**AUTHORS**

Gita V. Soraya^a,e^, Chathurika D. Abeyrathne^a,b,c^, Christelle Buffet^a,d^, Duc H. Huynh^b,c^, Shah Mukim Uddin^a,b^, Jianxiong Chan^a^, Efstratios Skafidas^b,c^, Patrick Kwan^a,c,^*, Stephen J. Rogerson^a,d,^*.

1. Department of Medicine, The University of Melbourne, Royal Melbourne Hospital, Victoria 3050, Australia.
2. Centre for Neural Engineering, The University of Melbourne, Carlton, VIC 3053, Australia.
3. Department of Electrical and Electronic Engineering, Melbourne School of Engineering, The University of Melbourne, Victoria 3010, Australia.
4. The Peter Doherty Institute for Infection and Immunity, Victoria 3000, Australia
5. Department of Biochemistry, Faculty of Medicine, Hasanuddin University, Makassar, South Sulawesi 90245, Indonesia.

* Contributed equally

* Corresponding authors: Stephen Rogerson: email sroger@unimelb.edu.au

Patrick Kwan: email patrick.kwan@monash.edu

**Optimization of Capture Antibody**

Capture antibody optimization was performed at the start of the study. Direct electrical parameters of output voltage (*V_out_*) and phase were used to determine changes in the sensors following incubation with high concentration recombinant *Pf*HRP2 protein in 1× PBS (50 ng/mL, 1.5 nM) against a human hemoglobin protein (HHb) negative (50 ng/mL, 0.8 nM), and blank PBS sensor (negative control). The human hemoglobin used as a negative was prepared by diluting human hemoglobin protein (powder form) in 1× PBS to make a stock solution of 4 mg/mL, which was then diluted down to the testing concentration using 1× PBS.

**
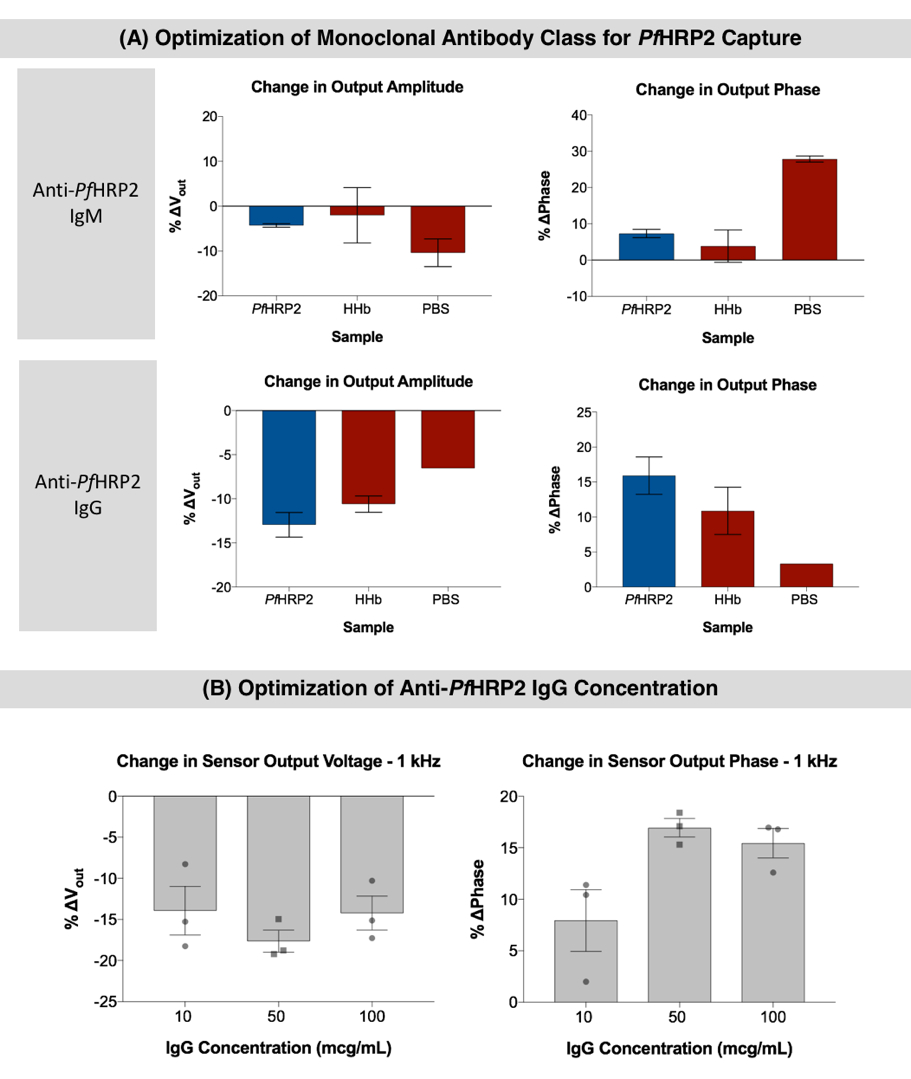
**

**Supplementary Figure 1.** Optimization of antibody class and concentration for *Pf*HRP2 capture. (A) Performance of IgM and IgG capture antibodies for *Pf*HRP2 detection. Results indicate that a higher magnitude of change in direct electrical outputs can be achieved using IgG MAb. Applied frequency 1 kHz, recombinant *Pf*HRP concentration and HHb concentration = 50 ng/mL. Figures show mean ± SEM. (B) Optimization of IgG concentration for *Pf*HRP2 capture. Results indicate 50 mcg/mL as the optimal concentration for *Pf*HRP2 detection, exhibiting the largest magnitude of change in both output phase and voltage.

**Modification of Blocking Protocol for Detection in Saliva**

To improve the specificity of the IDE sensor platform *Pf*HRP2 detection in saliva, the blocking solution was optimized to allow for optimum differentiation and minimum signal variation. Blocking solutions tested were: (1) 5% E = 5% ethanolamine, (2) 5%E + 2.5% GS = 5% ethanolamine mixed with 2.5% normal goat serum, and (3) 2.5%E + 2.5% GS = 2.5% ethanolamine mixed with 2.5% normal goat serum.

#
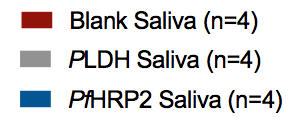

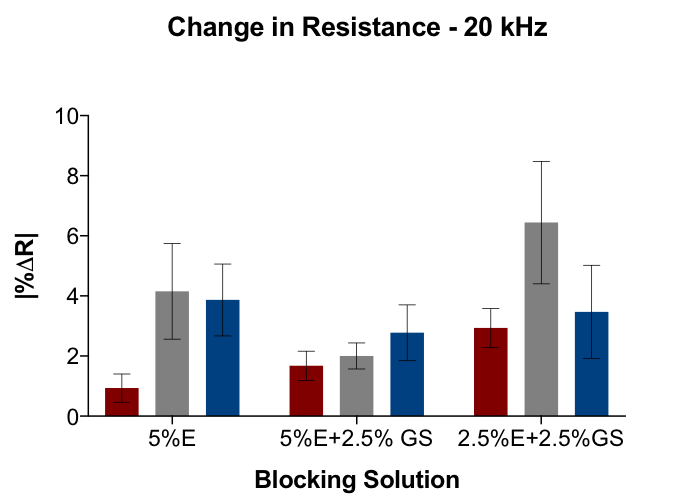


**Supplementary Figure 2.** Optimization of the blocking protocol to improve specificity of *Pf*HRP2 detection in saliva. Figures show mean ± SEM of the change in sensor resistance. **Incubation Time Optimization**

A longer incubation time was required for detection of culture-derived *Pf*HRP2 protein. The results indicate that incubation time increase to 2 hours resulted in the improved differentiation of *Pf*HRP2 protein obtained from CS2 culture.

**Supplementary Figure 3.** Optimal incubation time of culture-derived *Pf*HRP2 protein. Results indicate that incubation time of 2 hours allowed significant differentiation between positive *Pf*HRP2 (36 pg/mL, CS2 strain) spiked saliva against blank saliva. Applied frequency 20 kHz, figures show mean ± SEM, *n* ≥ 5 sensors per group, *p*-values obtained with Welch’s two-tailed T test.

**Statistical Quality Control of Sensors**

To illustrate the statistical method used to determine outliers, an example of the steps involved in elimination of faulty sensors are described below. Supplementary Table 1 shows the baseline impedance magnitude values of an experimental batch measurement of sensors. Using the PRISM ROUT test (Supplementary Figure 4A), outliers of the baseline measurements were detected. The results of the outlier test can be visualized in Supplementary Figure 4B Hence, sensors #7 and #23 were not used in the subsequent analysis of the experiment. This additional quality control step allowed elimination of sensor variations, which may have occurred during the fabrication (minor electrode damage during lift-off step) and functionalization (failure of silane growth on the sensor, failure of antibody attachment, or minor faults such as scratches) steps of the experiment.

**
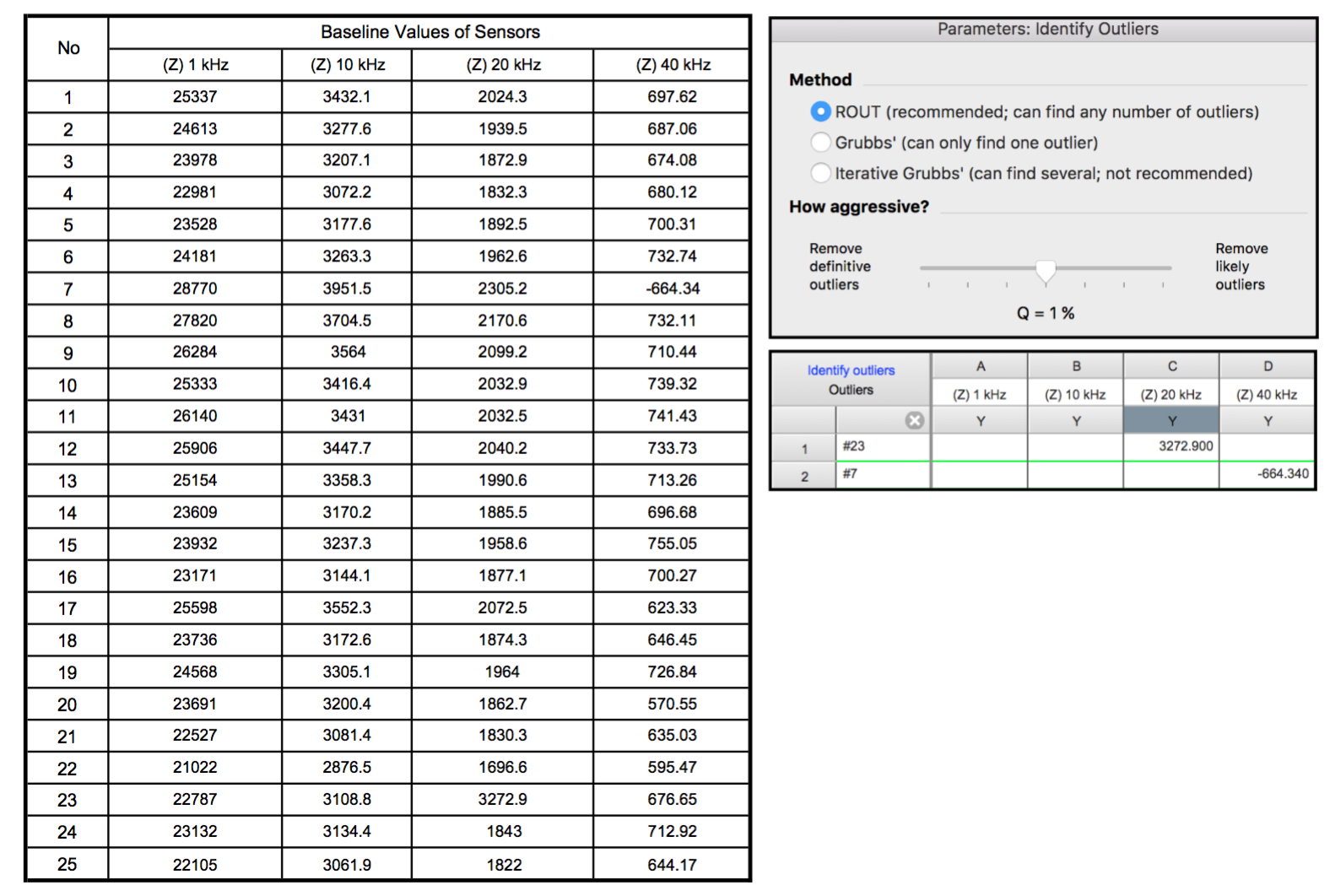
**

**Supplementary Table 1.** Example of baseline values of sensors. The table shows the baseline impedance magnitude values of 25 sensors in an experiment batch at the 4 different excitation frequencies.

**
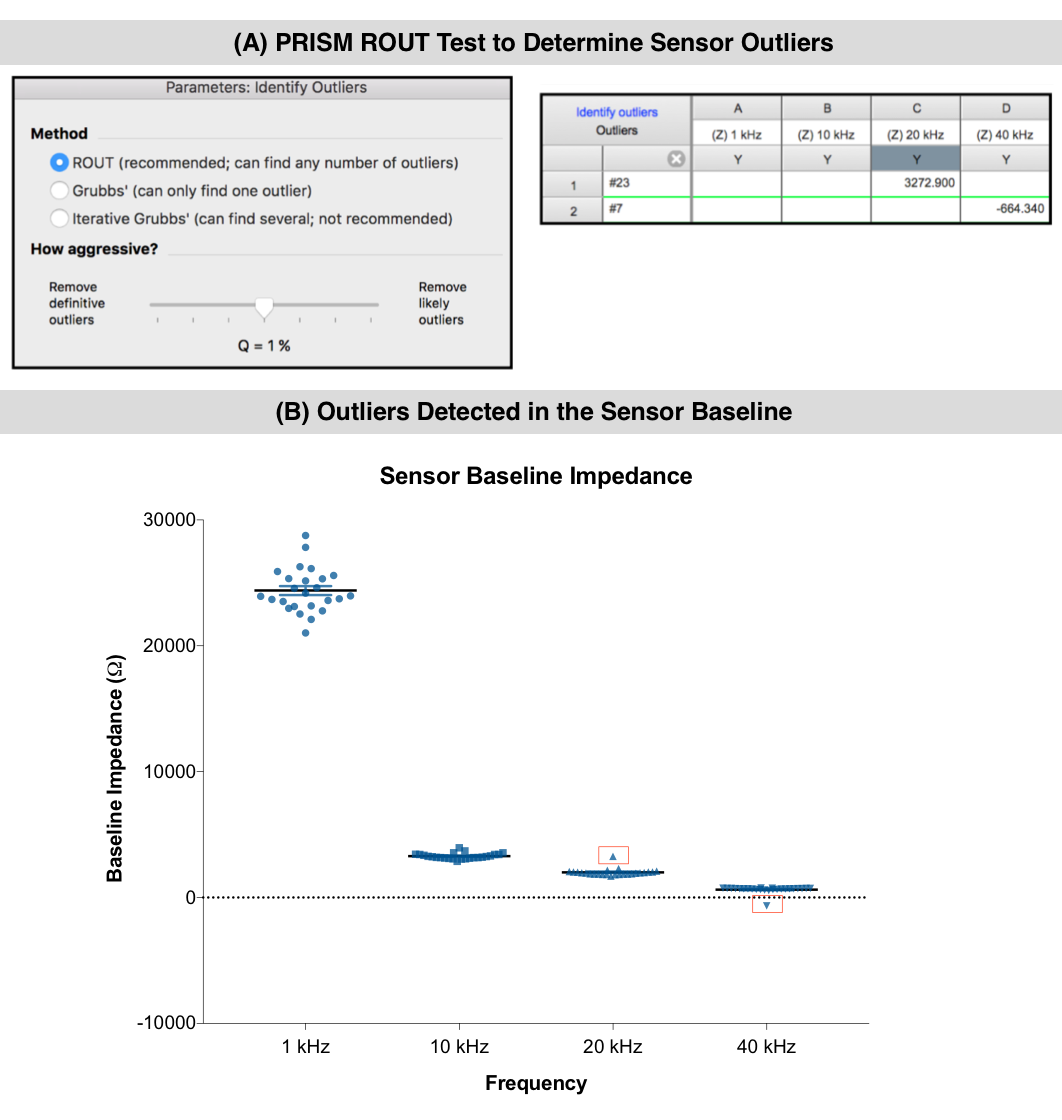
**

**Supplementary Figure 4.** (A) Prism ROUT Test set to determine outliers. Based on a Q value of 1%, the ROUT test enables better uniformity of the baseline by detecting sensors of high variation. (B) Depiction of the baseline impedance values. Graph illustrates the impedance magnitude (Z) of the 25 sensors across the experimental applied frequencies. Statistically detected outliers detected by the ROUT test are marked in red box.
